# Immune checkpoint analysis in ear cancer

**DOI:** 10.1186/s13005-023-00395-w

**Published:** 2023-11-06

**Authors:** M. Klein, E. Polgart, C. Hallermann, H. J. Schulze, F. Hölzle, K. Wermker

**Affiliations:** 1 Department of Oral & Maxillofacial Surgery, School of Medicine, University Hospital RWTH Aachen, Pauwelsstrasse 30, 52074 Aachen, Germany; 2 Hammer Straße 30, 48153 Münster, Germany; 3 Laboratory for Dermatopathology and Pathology Hamburg-Niendorf, Tibarg 7, 22459 Hamburg, Germany; 4 Department of Dermatology and Histopathology, Fachklinik Hornheide, Dorbaumstrasse 300, 48157 Muenster, Germany; 5 Department of Oral and Cranio-Maxillofacial Surgery, Klinikum Osnabrueck GmbH, Am Finkenhuegel 1, 49076 Osnabrueck, Germany

## Abstract

**Background:**

Among cutaneous squamous cell carcinomas, the ear (ecSCC) is one of the most common sites. Loco regional lymph node metastasis is found in six to eleven percent of cases, corresponding to increased metastasis compared to other sites. The aim of this study was to test the markers PD-L1, PD-1, CD4, CD8, and FoxP3 for suitability as prognostic predictive markers.

**Methods:**

Sixty-four patients with ecSCC were included in this study. The expression of immunohistochemical markers (PD-L1, PD-1, CD4, CD8, FOXP3) was correlated with retrospective clinic pathological parameters (lymph node metastasis, distant metastasis, lymph node metastasis during follow-up, disease progression, disease-specific death).

**Results:**

There was a correlation between increased disease specific death and a weak Foxp3 (*p* = 0.003) or reduced CD8 (*p* = 0.04). A PD-L1 expression > 1% was found in 39.1% of patients.

**Conclusion:**

The investigated markers (CD4, CD8, FoxP3, PD-1, PD-L1) seem overall rather inappropriate for prognostic evaluation in ecSCC. Only the correlation of disease specific death with CD8 or FoxP3 seems to be worth testing in larger collectives.

## Introduction

The cutaneous squamous cell carcinoma (cSCC) is the second most frequent skin cancer after the basal cell carcinoma in fair-skinned world population [1]. The risk for lymph node metastasis (LNM) of cSCC (excluding ear or lip) is 5%. Ear cutaneous squamous cell carcinoma (ecSCC) and squamous cell carcinoma of the lip (LSCC) however have higher metastasis rates of 8 to 10.5% [2, 3]. Other authors show even more aggressiveness in recurrence and metastasis rates of 10—25% in ecSCC and LSCC [4].

Immunohistochemical tumor markers could support the evaluation and prediction of tumor aggressiveness and metastasis rate. A tumor marker with such potential could be the programme cell death ligand (PD-L1) and programme cell death receptor (PD-1).

The PD-L1 pathway is physiologically essential for saving cells of overshooting immune reaction [5]. The PD-L1 molecule on cells can bind with the PD-1 receptor on t-cells (CD4, CD8) and stops the cells from destroying [6, 7]. The PD-1/PD-L1 pathway saves the tumor cells of destroying from CD8 cells and thus the tumor cell can survive [8]. High PD-L1 expression is correlated in cSCC with an increased metastasis rate [9]. Beside the prognostic effects of PD-1/PD-L1 pathway there is a therapeutic use of immune reaction in oncological therapy. Cemiplimab is an EMD approved PD-1 antibody against advanced cSCC [10]. In addition to the PD-L1/PD-1 pathway, other immune cells (CD8, CD4, FoxP3) appear to be exciting as tumor markers in the overall context. M. Klein et al. showed that PD-L1, PD-1, CD4, CD8 and FoxP3 were not useful as predictive outcome markers for LSCC. Nevertheless, the authors showed increased PD-L1 expression in the LSCC slides [11].

The aim of the study was to examine the tumor markers PD-L1 and PD-1 for their prognostic suitability in ecSCC. Special emphasis was to be placed on the quantitative expression analysis of PD-L1. In addition, immune cells of the tumor microenvironment (CD4, CD8, FoxP3) were to be evaluated for prognostic suitability and a particular focus should be on the prediction of LNM.

## Material and methods

### Patients

This study included patients with histologically saved ecSCC and an patient age of more than 18 years. Localizations of ecSCC were: retroauricular/posterior side, helix/lobules, cavum concae/anthelix/tragus and more than one region. All patients had a preoperative staging. All patients received staging according to the German guidelines for cutaneous squamous cell carcinoma (cSCC). For T1 / stage I this was ultrasound of the head an neck lymph drainage area. From T2 / stage II on all patients received CT of head and neck, CT thorax, ultrasound of the head and neck area and sonography of the abdomen.

Initial, primary therapy of all patients was surgery after case discussion in an interdisciplinary tumor board. In this study (matched pairs study population!) non-surgical cases were not included. Adjuvant radiotherapy was administered after tumor board decision according to guideline in cases with positive nodes (N +), close margin or R1/R2, perineural growth (Pn1) or invasion into blood vessels or lymph vessel (V1, L1).

Follow up was every 3 months in the first two years including ultrasound of the head an neck and CT or MRI scan once a year. For year 3 to 5 a follow-up interval of 6 months was chosen (also according to German guidelines).

Patients were excluded from analysis with incomplete data sets and other head and neck cancers. Tumor characteristics and follow-up data were collected retrospectively from the authors’ institutional database.

### Immunohistochemistry

The IHC implementation and analysis were performed at the dermatohistopathologic laboratory of the Fachklinik Hornheide (Muenster, Germany). This study method oriented towards the study of M. Klein et al. [11].

For the first overview on tumor and peritumoral environment every tissue sample (primary tumor, no biopsy) was analyzed with HE staining. The used microscope was the Olympus BX51 microscope (Hamburg, Germany) with magnification of 400 × . All pictures were made with the Olympus UC30 microscope camera (Hamburg, Germany) and the Olympus cell sense entry programme (Hamburg, Germany) was used.

### Immunohistochemistry CD4, CD8, FOXP3 and PD-1

Formalin Fixed and Paraffin embed (FFPE) ecSCC were cut in slices (4 μm) and evacuated on coated slides. Reaction with primary antibody were performed in the Autostainer Plus (Dako REAL DETECTION SYSTEM K5005, Glostrup, Denmark). Table 1 gives an overview of the primary antibodies used and their application.

**Table 1:** Shows an overview of used primary antibody, pretreatment, dilution and exposure time. Footnotes: 1 Leica, Newcastle Upon Tyne, United Kingdom, 2 Agilent/Dako, Glostrup, Denmark, 3 Abcam, Cambridge, United Kingdom, 4 CELL MARQUE, Rocklin, USA

Antigene	Clone	pretreatment	Dilution	Exposure time	Manufactor
CD4	Novocastra tm Liquid Mouse Monoclonal Antibody CD4 Product Code: NCL-L-CD4-368	heat, ph 9	1:10	25 min	Leica1
CD8	FLEX Monoclonal Mouse Anti-Human CD8 Clone c8/144B	heat, ph 9	undiluted	25 min	Agilent/Dako2
FoxP3	anti-FOXP3 antibody [236A/E7] ab20034	heat, ph 9	1:50	25 min	Abcam3
PD-1	MRQ-22;	heat, ph 9	undiluted	20 min	CELL MARQUE4

The tumor slices were then reacted (exposure time 15 min) with a secondary antibody (REAL Link Biotinylated secondary antibody (AB2)). After that the tissue slices were incubated (exposure time 15 min) with Dako Real Streptavidin Alkaline Phosphatase (AP) (Glostrup, Denmark) and reacted (8 min) with chromogen (Dako RED Chromogen, Glostrup, Denmark). As a nucleus counterstain all slices were colored with Haematoxylin (exposure time 8 min).

All slices were dehydrated with increasing alcohol concentrations (70%, 90% and 100%), processed with xylol. All colored slices were covered with Coverslip tape.

The analysis were performed by two independent investigators. Anti-CD4, anti-CD8, anti-FOXP3 and anti-PD-1 were analyzed in a semiquantitative analysis mainly in the subepithelial compartment in a hot spot analysis. Each cut was counted in five high power fields with 50 cells each, totalling 250 cells.

### Immunohistochemistry for PD-L1

Formalin Fixed and Paraffin embed (FFPE) ecSCC were cut in slides (4 μm) and evacuated on coated slides. The sections were then pretreated using the pretreatment system PT link Dako (Glostrup, Denmark) at a pH of 6 and subsequently stained with the PD-L1 panel (PD-L1 ICH 22C3 pharmDx, Agilent/Dako, Glostrup, Denmark) in the Autostainer link 48 (Agilent/Dako, Glostrup, Denmark). Finally, the material was covered with a coverslip.

In addition, a color control was performed. Skin was used as a negative control and placenta as a positive control.

For analysis of anti-PD-L1 the recommendation of the PD-L1 IHC 22C3 pharmCx Interpretation Manual (Agilent/Dako, Glostrup Denmark) was used.

Two independent investigators analyzed the tissue samples with the tumor proportion score (TPS). PD-L1 expression was categorized into three groups. First no expression = 0% (0 pts.); second positive weak to moderate = 1% to 49% (1 pt.); and third positive strong with expression of ≥ 50% (2 pts.). Besides, expression and intensity of PD-L1 staining were combined to generate a PD-L1 score summing up the above-given points. The PD-L1 score therefore had a theoretical range from 0 to 5 points.

For PD-L1, intensity of staining was analyzed in an ordinal scale: none (0 points [pts.]), weak (1 pt.), moderate (2 pts.) and strong (3 pts.) colouring.

### Statistical analysis

All statistical analyses were conducted by a statistician using the Statistical Package for Social Sciences (SPSS) version 22.0 for Windows® (SPSS Inc., Chicago, Illinois, USA).

Data were tested for normal distribution with the Kolmogorov–Smirnov test. The t test was used for normally distributed variables. For categorical variables, Fisher's exact test and the chi-square test were applied. For metric parameters, the Kruskal–Wallis H test or Mann–Whitney U test were used as non-parametric tests for not normally distributed data. All tests were two-sided. Survival time periods (time from first diagnosis until event; data on patients without event were censored at the last follow-up) were calculated using the Kaplan–Meier method, and group differences were analyzed using the log-rank test.

A special focus of our study was set on the predictive testing of the markers (CD4, CD8, FoxP3 PD-1, PD-L1) for the risk of LNM. The matched pair approach is suitable for this purpose. The matched pair approach can minimise the influence of known risk factors. For this purpose, two homogeneous groups were formed. In one group were patients with LNM + (*n* = 32) and in the other group patients with LNM- (*n* = 32). The groups were matched with the following parameters with the smallest possible differences: gender, age, immunosuppression, comorbidities, primary localization, T-stage, tumor thickness, grading and perineural growth (Pn). Comparability and homogeneity of this stratification were checked statistically using matched pairs analysis.

Randomization and blinding were used for the IHC analysis. The researcher analyzing the IHC staining did not know which group the patient belonged to.

The subgroups were determined by dividing the patients according to primary tumor location, American Joint Committee on Cancer stage (AJCC stage) and lymph node metastasis.

## Results

### Overview of the study population

The following is an overview of the cohort of ecSCC patients: Of all included patients (*n* = 64), 59 were male and 5 female. Clinical data were collected: T1 (*n* = 8), T2 (*n* = 9), T3 (*n* = 39), T4 (*n* = 8), N0 (*n* = 37), N1 (*n* = 18), N2a (*n* = 7), N2b (*n* = 2), M (*n* = 0), G1 (*n* = 17), G2 (*n* = 31), G3 (*n* = 16), Pn 1 (*n* = 4), Linv (*n* = 3), N + (during follow-up; *n* = 20), distant metastasis (DM) during follow-up; *n* = 3).

The ASA (American Association of Anesthesiologists) score was determined to estimate patient constitution: ASA 1 (*n* = 1), ASA 2 (*n* = 21), ASA 3 (*n* = 40), ASA 4 (*n* = 2).

In addition, the immune suppression was evaluated: no immunosuppression (*n* = 51), mild immunosuppression (*n* = 6) and strong immunosuppression (heart transplantation; *n* = 1).

The recurrence rate was also determined. There were no recurrences in 44 of the patients, one recurrence in 15 patients, two recurrences in 4 patients and three recurrences in one patient. Disease-specific death was seen in 13 patients.

### Analysis of the markers CD4, CD8, FoxP3, PD-1 and PD-L1

**Fig. 1:**
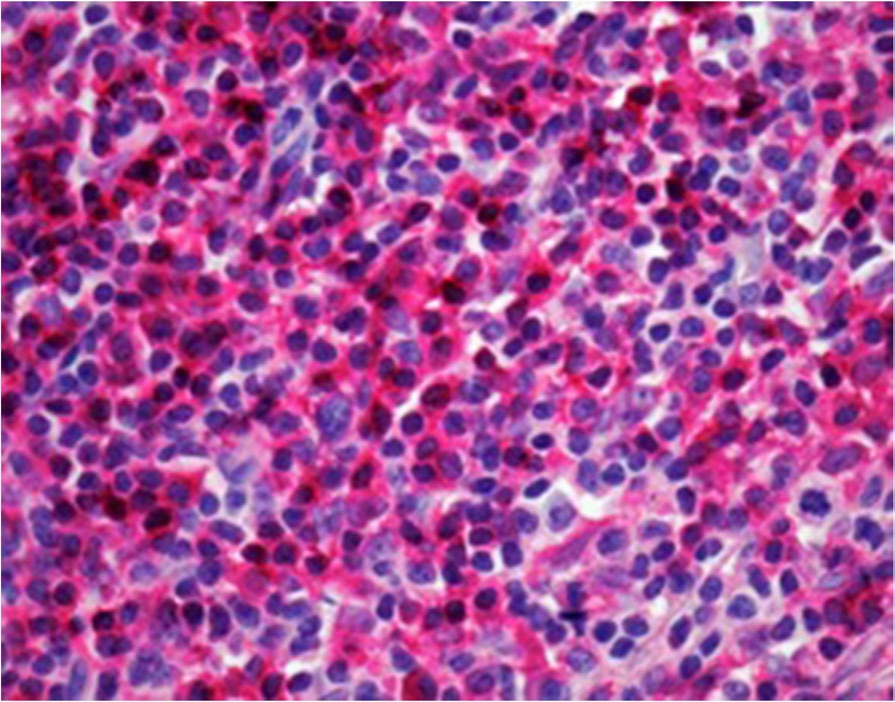
Shows immunohistochemically stained CD4 + t-cells in the tumor microenvironment (magnification 400 × ; tissue Sect. 4 μm)

**Fig. 2:**
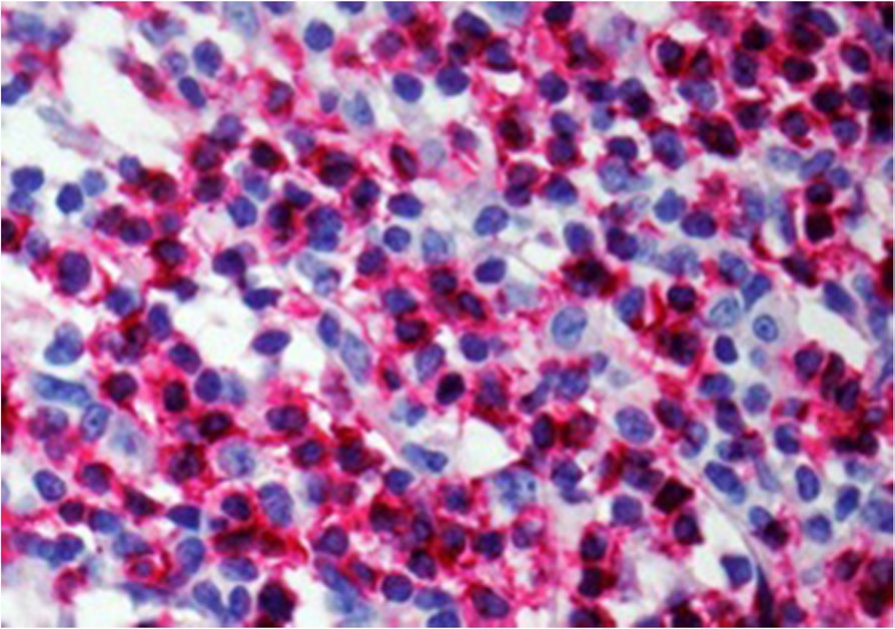
Shows immunohistochemically stained CD8 + cytotoxic t-cells (magnification 400 × ; tissue Sect. 4 μm)

**Fig. 3:**
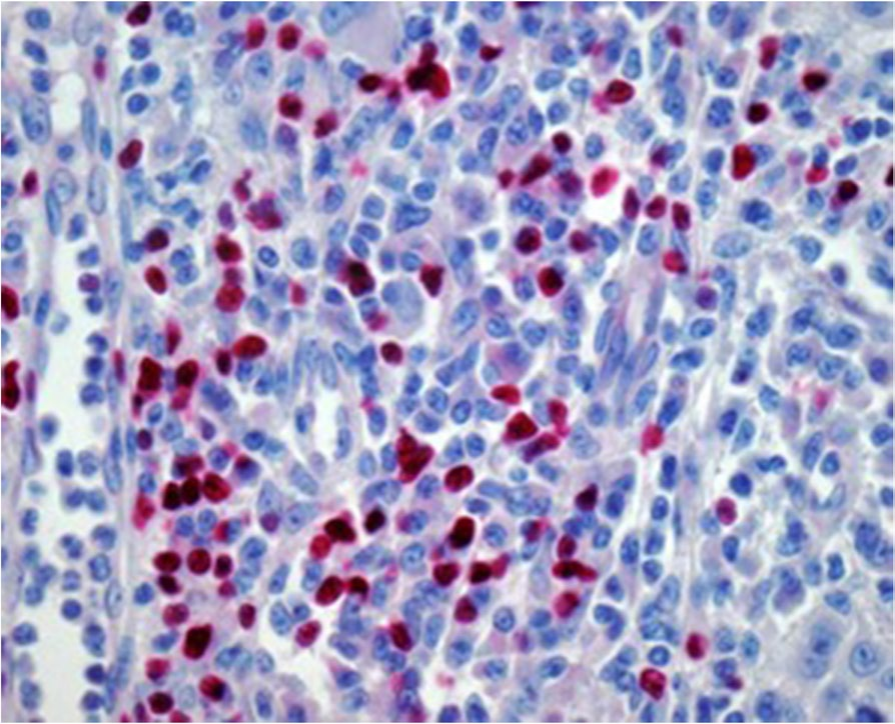
Shows immunohistochemically stained PD-1 + (magnification 400 × ; tissue Sect. 4 μm)

**Fig. 4:**
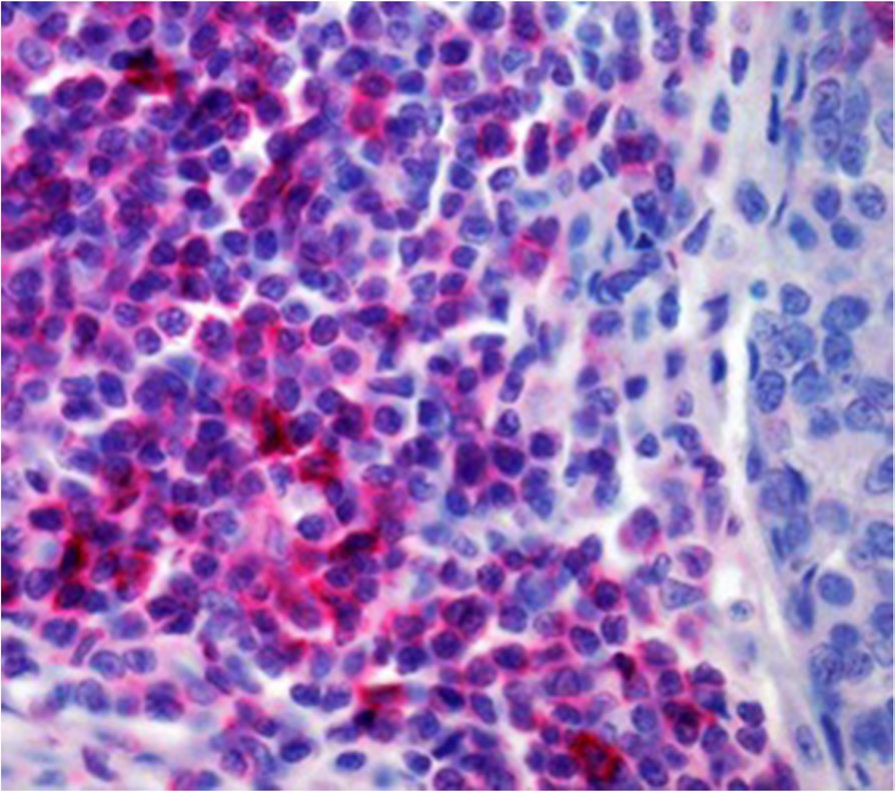
Shows immunohistochemically stained Foxp3 + regulatory t-cells (magnification 400 × ; tissue Sect. 4 μm)

**Fig. 5:**
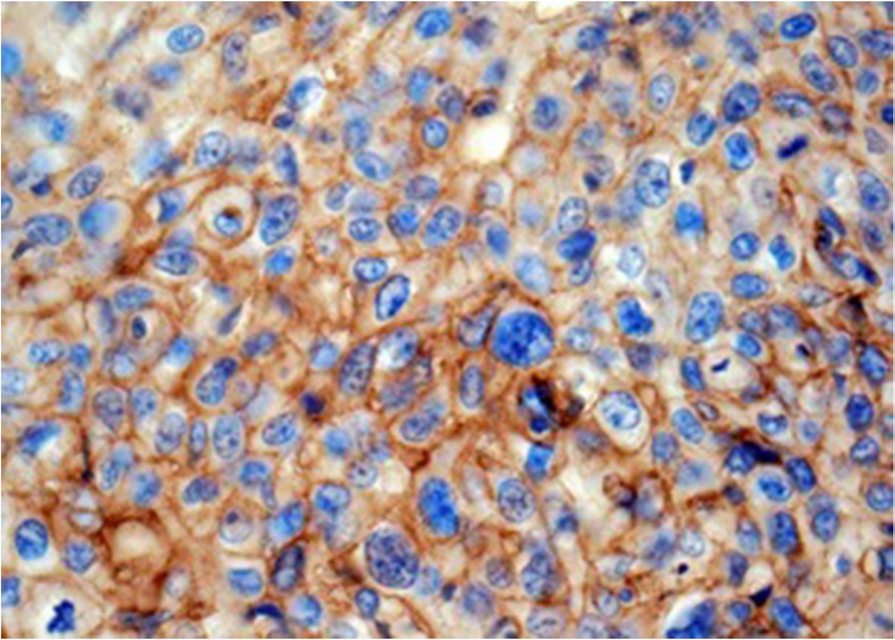
Shows immunohistochemically stained PD-L1 + squamous cell carcinomas of the ear (ecSCC; magnification 400 × ; tissue Sect. 4 μm)

In the following, the markers CD4, CD8, FoxP3, PD-1 and PD-L1 are presented correlated with clinical aggressiveness and outcome parameter. Aggressiveness characteristics (LNM, distant metastasis (DM)) and outcome prediction characteristics (LNM during follow-up, disease progression, disease-specific death, relapse) were determined for each marker. Figures 1, 2, 3,  4 and 5 show representative staining examples.

### CD4 expression

There was no significant correlation of CD4 and aggressiveness characteristics (LNM, DM) and outcome prediction characteristics (LNM during follow-up, disease progression, disease-specific death, local relapse).

### CD8 expression

No significant correlation of CD8 expression and LNM, DM, LNM during follow up, disease progression or local recurrence could found.

**Fig. 6:**
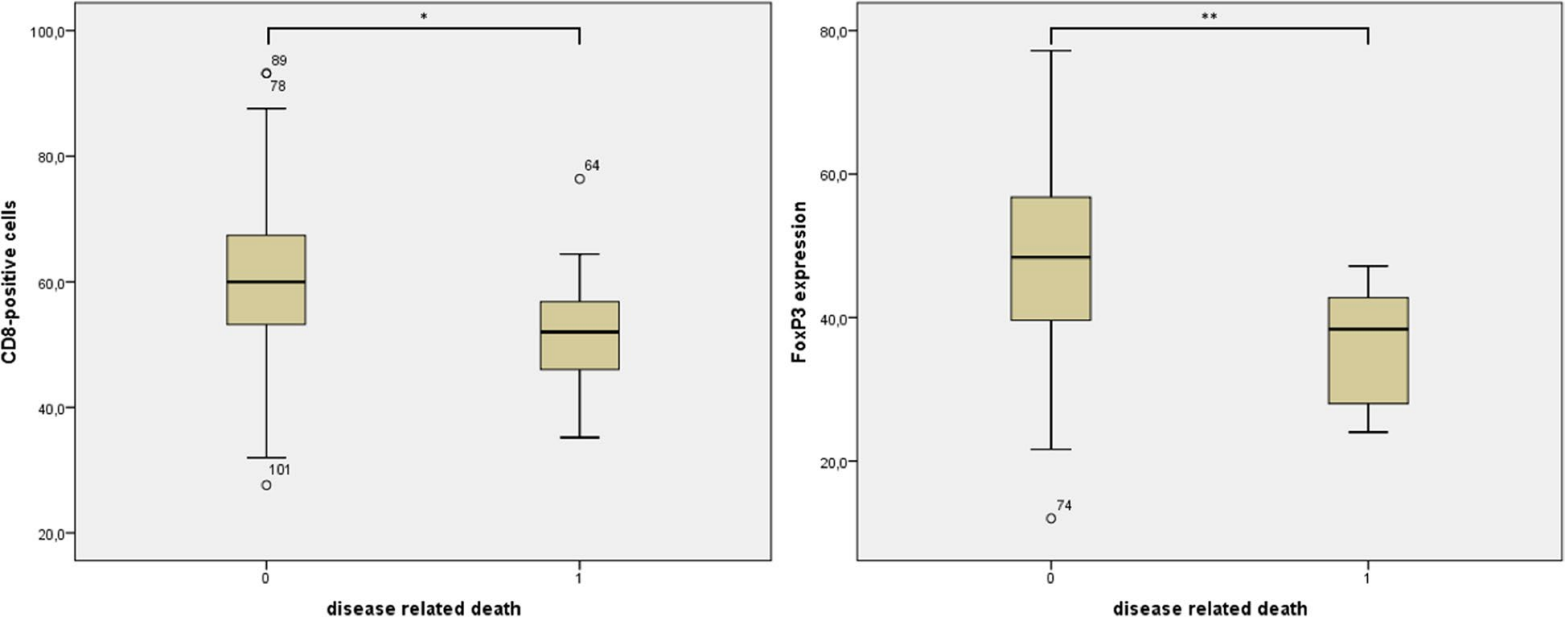
Shows a boxplot diagram of the disease-specific survival and the expression of CD8 (left side). On the right side, disease-specific survival is plotted against FoxP3 expression. Increased disease-specific survival correlates significantly with decreased CD8 expression (*p* = 0.04) and decreased FoxP3 expression (*p* = 0.003)

A significant correlation (*p* = 0.04; Fig. 6 left side) could be found between low CD8 expression and disease specific death.

### FoxP3 expression

There was no significant correlation between FoxP3 expression and LNM during follow up, disease progression or local recurrence. Even if not significant, some trends for FoxP3 could be presented. Reduced FoxP3 expression showed a tendency for LNM (*p* = 0.066) and DM (*p* = 0.089). However, the results failed to reach significance.

There was a correlation between decreased FoxP3 expression and increased disease specific death (*p* = 0.003; Fig. 6 right side).

### PD-1 expression

No correlation of PD-1 expression with LNM, LNM during follow-up, disease progression, disease-specific death or local recurrence could be ascertained.

In addition, the analysis shows only a trend (p = 0.081) between low PD-1 expression and DM. However, the results failed to reach significance.

### PD-L1 expression

First, PD-L1 expression and intensity were determined. 39 patients (60.9%) showed no PD-L1 expression, 22 patients (34.4%) presented with moderate PD-L1 expression and 3 patients (4.7%) had strong PD-L1 expression. There was no significant difference between PD-L1 expression and LNM (*p* = 0.383) or DM (*p* = 0.365).

Seventeen patients (26.6%) had a low intensity, 5 patients (7.8%) presented a moderate intensity and 3 patients (4.7%) had strong PD-L1 intensity. There were no significant difference between PD-L1 intensity and LNM groups (*p* = 0.557) or DM (*p* = 0.569).

The PD-L1 expression was not correlated with LNM during follow up, disease progression or local recurrence.

In contrast, there was only a trend (*p* = 0.074) of disease specific death and PD-L1 expression.

### Summary of the results

In the following, the significances of the examined markers with the respective clinically pathological known risk factors are summarized once again: A significant correlation could be found between disease specific death and low CD8 expression (*p* = 0.04) and also for a decreased FoxP3 expression (*p* = 0.003).

## Discussion

### Strengths and weaknesses of study design

There are some strengths and weaknesses in the study design of immune checkpoint analysis in ecSCC. The immune checkpoint marker PD-L1 and PD-1 and the marker of tumor microenvironment (CD8, CD4, FoxP3) were analyzed in a relevant number of ecSCC. The matched pairs approach could have generated a selection bias. Other covariables which could influence the result would have been restricted in inclusion criteria to minimize the bias.

The matched pair can contrast other known risk factors with similar characteristics and control them. No comparable study analyzing ecSCC by balancing N + and N- patient groups using a matched pairs study design has been performed. Every marker will be discussed alone in the following.

Klein et al. show in LSCC that there where similar result to this study. There were no correlation between the marker PD-L1, PD-1, CD4, CD8 and Foxp3 and clinic pathological outcome factors. The LSCC has got also a higher rate for cervical metastasis compare with other cSCC of other localization [11]. Because of the similar method the study is a good comparability.

### CD4

The prognostic utility of CD4 has been described ambiguously in the literature. Similar results to our own results of the prognostic relevance of CD4 in the tumor microenvironment were found in the study by Balermpas et al. No significant association was found between CD4 expression and local recurrence, progression-free survival, overall survival or distant metastasis [12]. In LSCC as a high risk localization of cSCC, there was also no prognostic benefit of CD4 [11].

In contrast to these findings, Nguyen et al. show different results. The study team demonstrated a correlation between CD4 expression and better overall and disease-specific survival. They suggested CD4 as a potential biomarker for HNSCC (Nguyen et al., 2016). Badoual et al. were able to show that an increased CD4 level leads to better locoregional control and overall survival [13]. The debate on the clinical benefit of CD4 in ecSCC is ongoing.

### CD8

CD8 expression appears to have a prognostic benefit compared to CD4 expression. The significant correlation between low CD8 expression in ecSCC and disease specific death is perhaps an indication of the poorer immune defense in the tumor and the reduced immune system of the patient.

Balermpas et al. showed that there is a correlation between a certain amount of infiltrating CD8 + t-cells and prognostic outcome [12]. Many studies in HNSCC showed a better clinical outcome when there was increased CD8 + expression [14]. In a comparison with HNSCC, there was better overall survival in oropharyngeal, oral cavity and laryngeal cancer with increased CD8 + T cells [15].

A comparison can also be made with LSCC. In contrast to the results shown here, CD8 seems to have a prognostic benefit in ecSCC, at least in terms of disease-specific survival. These results could not be demonstrated for LSCC [11].

### FoxP3

It has been proposed in the literature that tumor cells may attract regulatory t-cells and establish local immunosuppression. This keeps the immune cells from destroying the tumor [16–18].

Kindt et al. showed in head and neck cSCC that an increased FoxP3 number in the stromal compartment correlated with significantly better patient recurrence free and overall survival [19]. A meta-analysis by De Ruiter et al. showed that a high tumor infiltration of FoxP3 T cells is associated with a better clinical outcome in HNSCC [14]. Our data show that ecSCC behaves similarly to other squamous cell carcinomas of the head and neck region. Foxp3 thus has a high potential as a predictive outcome marker. Also, the trends of FoxP3 with LNM and DM seem worthy of review and control in further studies.

### PD-1

The expression of PD-1 in ecSCC does not appear to have prognostic significance comparable to the analysis in LSCC [11].

There is disagreement in the literature about the prognostic significance of PD-1 expression. Balermpas et al. analyzed the impact of PD-1 expression on HNSCC. The team showed that there was no prognostic effect [20]. Schneider et al. showed different results. The team analyzed PD-1 expression in HNSCC and showed that PD-1 expression could be used as a prognostic marker. A correlation with overall survival (*p* = 0.004) and disease-free survival (*p* = 0.001) was shown [21].

The trend of low PD-1 expression with increased risk of DM seems worthy of review in a larger collective for ecSCC.

### PD-L1

There are factors that influence the reproducibility of PD-L1 expression. Liu et al. showed that several factors influence the PD-L1 expression. The team cited the heterogeneity of the tumor, different methods in marker protocols, size and position of the biopsy and defined that it is only a snapshot of the PD-L1 expression of the tumor [22].

Similar results were also shown in a study by De Meulenaere et al. The authors stated that the reasons for the PD-L1 variations are different marker assays, cut-off values and heterogeneity of PD-L1 expression. It is possible that therapy (systematic chemotherapy or radiation) has an impact on PD-L1 expression. Perhaps it would be beneficial to analyze PD-L1 expression at different time points, e.g. at initial diagnosis, during therapy and during tumor progression [23].

Likewise, due to the similar tumor aggressiveness and increased LNM compared to other cSCC, a comparison can be made with LSCC.

In the study of M. Klein et al. more than the half of the analyzed collective (56.9%) show a PD-L1 expression > 1%. This underlines that the tumor biology of the both risk localization of cSCC is comparable [11].

In addition to the comparison with high risk localizations, a comparison with risk factors for LNM can also be carried out. Slater et al. also worked with Dako's TPS and found a positive correlation between PD-L1 expression and pathological findings that were related to risk for LNM: large tumor diameter, higher histological grading and vertical tumor thickness [9].

Even though PD-L1 expression does not appear to be prognostically applicable in ecSCC, 39.1% of patients have PD-L1 expression > 1%. Nevertheless, therapeutic options with immune checkpoint inhibitors could be derived from this for advanced tumor stages.

For follow-up studies, it should be noted from the point of view of therapeutic usability and therapy with immune checkpoint inhibitors that the focus should be placed on advanced stages, since here the indication for therapy and PD-L1 diagnostics is given.

The Keynoe-012 study by Seiwert et al. from 2016 shows the efficacy of pembrolizumab in metastatic and/or relapsed HNSCC with a PD-L1 expression of ≥ 1%. Based on the study, the PD-1 inhibitor pembrolizumab was approved in the USA for HNSCC [24]. Cemiplimab, a PD-1 inhibitor with FDA and EMA approval, has a response rate of 44% in locally advanced cSCC. In metastatic patients, the overall response rate is 49.2% [25]. Future studies should also focus on response rates in different cSCC locations and whether differences can be shown.

## Conclusion

The investigated markers (CD4, CD8, FoxP3, PD-1, PD-L1) seem overall rather inappropriate for prognostic evaluation in ecSCC. Only the correlation of disease specific death with CD8 or FoxP3 seems to be worth testing in larger collectives.

## Data Availability

The datasets (without patients details) used and analyzed during the current study are available from the corresponding author on reasonable request.
